# Features of peritoneal dendritic cells in the development of endometriosis

**DOI:** 10.1186/s12958-023-01058-w

**Published:** 2023-01-13

**Authors:** Zheng Qiaomei, Wu Ping, Zhao Yanjing, Wang Jinhua, Chen Shaozhan, Chen Lihong

**Affiliations:** 1 Department of Obstetrics and Gynecology, Fujian Key Laboratory of Precision Medicine for Cancer, the First Affiliated Hospital, Fujian Medical University, 20 Chazhong Road, Fuzhou, Fujian 350005 People’s Republic of China; 2 Department of Gynecology, National Regional Medical Center, Binhai Campus of the First Affiliated Hospital, Fujian Medical University, 999 Huashan Road, Fuzhou, Fujian 350212 People’s Republic of China; 3 Department of Pathology, the First Affiliated Hospital, Fujian Medical University, 20 Chazhong Road, Fuzhou, Fujian 350005 People’s Republic of China; 4 Department of Surgery, 92403 Military Hospital, Fuzhou, Fujian 350015 People’s Republic of China

**Keywords:** Endometriosis, Peritoneal dendritic cells, DCs maturation, Immature DCs, Mature DCs

## Abstract

**Background:**

Emerging evidence of immunological dysfunction have been described in endometriosis. Dendritic cells (DCs), one of the main antigen-presenting cells, are specialized in the initiation and modulation of the adaptive immune response. Emerging studies demonstrated both endometrial and circulating differences in DCs populations in women with endometriosis. However, the role and mechanism of peritoneal DCs in endometriosis is still unclear. The present study was undertaken to explore the features of peritoneal DCs in the pathogenesis of endometriosis. This study is beneficial to further clarify the cause of endometriosis and provide a new insight into the medical treatment for endometriosis.

**Methods:**

The study included 12 women with endometriosis and 11 women without endometriosis. The C57BL6 mouse model of endometriosis was established by intraperitoneal injection of endometrial segments. The peritoneal DCs of endometriosis patients and mouse models were analyzed by fluorescence associated cell sorting (FACS) examination.

**Results:**

Increased cell density of peritoneal DCs were observed in endometriosis patients. Moreover, the proportion of mature DCs (mDCs, CD80^high^CD1a^low^ cells) in the peritoneal DCs was lower whereas the proportion of immature DCs (iDCs, CD80^low^CD1a^high^ cells) was increased in endometriosis patients. Similarly, the cell density of peritoneal DCs in murine models increased immediately after the injection of endometrial tissues and reached the highest level at 14 days. In addition, the proportion of mDCs (CD11c^high^CD80^high^ cells) in the peritoneal DCs decreased immediately after the injection of endometrial tissues and then increased with the time until 42 days, but still lower than the control group. In contrast, the proportion of iDCs (CD11c^high^CD80^low^ cells) in the peritoneal DCs showed the opposite dynamic changes. However, after treated with LPS, the mDCs proportion was significantly increased, leading to lower volume and weight of the endometriosis lesions.

**Conclusions:**

Increased level of peritoneal DCs facilitated the pathogenesis of endometriosis lesions, especially in the early stage of the disease. Furthermore, peritoneal DCs maturation played an important role in the development of endometriosis.

## Background

Endometriosis, characterized by the presence of functional endometrium tissues outside the uterus, is known as an inflammatory disease [1]. Although endometriosis is a benign gynecology disease, it affects around 10% of childbearing-age women, resulting in dysmenorrhea, dyspareunia, chronic pelvic pain, infertility and decreased quality of life [2]. Several hypotheses, including the retrograde menstruation and implantation theory, the induction theory, and the Müllerian remnants theory, have been proposed to explain the pathogenesis of endometriosis [3]. However, there is no medical or surgical cure for endometriosis due to the complex and multifactorial nature of the disease. The recurrence rate of endometriosis is more than 20% within 2 years after surgery [4]. Up to now, medical therapies for endometriosis mainly focused on altering sex steroid hormones, not only require medical treatment until menopause, but also resulting in many side effects [5, 6].

While the pathogenesis of endometriosis is poorly understood, it is supposed that the immune system plays a role in the development of endometriosis, facilitating the establishment and persistence of endometriotic lesions after displacement of endometrial tissue into ectopic locations within the peritoneal cavity [7, 8]. Enhanced understanding of immune mechanisms occurring at the site of ectopic endometriotic lesion would therefore provide inestimable insight into the pathogenesis of endometriosis. Specifically, endometriotic lesions display altered immunity profiles compared to normal endometrium [9]. As reported, transcriptomic profiling revealed significantly differential expression of immune-inflammation genes in ectopic tissues compared with control endometrium [10]. Moreover, Suryawanshi et al. revealed that endometriotic lesions from patients with endometriosis possess a distinct immune microenvironment resembling a tumor-like inflammatory profile [11]. Emerging evidence of immunological dysfunction, including the increased number of immune cells and changes in immunological function, have been described in endometriosis [12, 13]. Therefore, focusing on the inflammatory immune response in endometriosis may lead to a better therapy for the disease.

Dendritic cells (DCs), one of the main antigen-presenting cells (APCs), play a crucial role in the immune response due to their functions as mediators between the innate and adaptive immunity and their unique ability to modulate the adaptive response [14, 15]. Upon pathogen recognition, DCs obtain the ability to capture, process, present antigens, and produce cytokines, facilitating the following pathogen-specific effector T cells differentiation and activation, and then resulting in the ongoing immune responses [16]. Apart from that, DCs can also promote self-tolerance by secreting tolerogenic cytokines, leading to the break of tolerance and the pathogenesis of autoimmune diseases [17, 18]. As reported, the role of DCs were altered in endometriosis. Hey-Cunningham et al. confirmed both endometrial and circulating differences in DCs populations in women with endometriosis, with disease stage-specific relationships in the endometrium [19]. However, the role of DCs in the development of endometriosis lesions in murine models was inconsistent [20–22]. So, this study was undertaken to explore the role of DCs in the pathogenesis of endometriosis.

Traditionally, DCs are divided into two major subsets: plasmacytoid DCs and myeloid DCs (also known as conventional DCs and classical DCs) [23]. Additionally, DCs have two basic functional stages: immature DCs (iDCs) and mature DCs (mDCs) [24]. Schulke L et al. proved that both iDCs and mDCs populations were altered in the eutopic and ectopic endometrium of endometriosis patients [25]. Moreover, emerging studies demonstrated that DCs in peritoneal fluid (peritoneal DCs) are susceptible to pro-endometriotic changes by inhibiting iDCs from their maturation [26, 27]. Therefore, our hypothesis is that the maturation of peritoneal DCs participates in the pathogenesis of ectopic endometriosis lesions. The present study was undertaken to explore the features of peritoneal DCs in endometriosis patients. Furthermore, we investigated the dynamic changes of peritoneal DCs maturation in murine endometriosis models and proved the crucial role of peritoneal DCs maturation in the pathogenesis of endometriosis. This study is beneficial to further clarify the cause of endometriosis and provide a new insight into the medical treatment for endometriosis.

## Methods

### Patients and samples

The peritoneal DCs in endometriosis group were obtained from 12 women with endometriosis (mean age 31.17 ± 0.8862 years [range 27–37]), of whom 8 had AFS stage III disease and 4 had AFS stage IV disease. The peritoneal DCs in control group were obtained from 11 women without endometriosis (mean age 30.27 ± 1.184 years [range 24–37]). The endometriosis group consisted of women who was visually diagnosed during the laparoscopy for endometrioma and then ascertained by pathological examination. The control group consisted of women who had surgery for other benign ovarian cysts (exclusion of endometriosis by laparoscopic surgery and pathological examination). All samples were taken at the proliferative phase of menstrual cycle. Peritoneal fluids were obtained right after penetrating into peritoneum during operations. Peritoneal cavity was douched with 20 ml normal saline at first. Then, 15 ml peritoneal fluid was aspirated, processed and used for the following fluorescence associated cell sorting (FACS) examination. All of the participants had regular menstruation and received no hormonal therapy for at least 6 months before the study, chosen from the Department of Obstetrics and Gynecology, First Affiliated Hospital of Fujian Medical University, from February 2021 to November 2021. Informed consents were obtained from all participants prior to surgery. The Ethics Committee of the First Affiliated Hospital of Fujian Medical University approved the study (approval number: 2021[017]).

### Mouse model of endometriosis

The C57BL6 mice in the present study were obtained from Beijing HFK Bioscience Company (Beijing, China). All procedures were conducted in accordance to the Animal Care and Use Committee of Fujian Medical University (Fujian, China). All mice were caged for 2 weeks to acclimatize to the environment, during which time the estrous stage was monitored daily. Mice with normal estrous cycles were used in the following experiments. The mouse model of endometriosis was established by intraperitoneal injection of endometrial segments as described previously [28]. The donor mice were initially treated with estradiol benzoate (3 μg/mouse, Aladdin, Shanghai, China) for 7 days. Then, the donor mice were sacrificed and their uteri were removed in a petri dish containing warm sterile phosphatebuffered saline (PBS, PH 7.2–7.4). Each uterine horn was split longitudinally and carefully disrupted into small fragments smaller than 1 mm. Then, the fragments were injected intraperitoneal into recipient mice using a 1-ml syringe and a 25-guage needle. All mice received endometrial fragments i.p. injection were randomly distributed into endometriosis group and LPS (lipopolysaccharide, 25 μg/mouse, which can initiate DCs maturation) group [29]. To distinguish the changes of DCs caused by intraperitoneal injection, mice in the control group received an peritoneal lavage of PBS. Three mice in each group were sacrificed at each time point (3, 7,14,21,28,42 days) for the following experiments.

After sacrificed, peritoneal cells were harvested from the model by injecting and shaking 5 ml of ice-bath PBS buffer. After peritoneal lavage harvest, 3 ml peritoneal fluid was heparinized and centrifuged for 300 g × 10 minutes. Then, the cell pellet was re-suspended in PBS and centrifuged for 900 g × 30 minutes. Thereafter, the red blood cells were removed by lysising with NH_4_Cl lysing buffer. Cells were then washed with PBS and re-suspended in FACS buffer for the following FACS analysis. All animal experiments were approved by the Laboratory Animal Ethics Committee of Fujian Medical University (IACUC FIMU 2022-NSFC-0248).

### FACS examination

The DCs in the endometriosis patients were characterized as the following: CD80^high^CD1a^low^ cells for mDCs and CD80^low^CD1a^high^ cells for iDCs [8, 24]. Cells in the peritoneal fluid of patients were incubated with the following antibodies: anti-CD80-PE and anti-CD1a-APC (Miltenyi Biotec), and analyzed with the use of FACS. Similarly, cells in the peritoneal fluid of mice were incubated with the following antibodies: anti-CD80-PE and anti-CD11c-APC (Miltenyi Biotec). In the murine endometriosis model, CD11c^high^CD80^high^ cells were defined as mDCs, and CD11c^high^CD80^low^ cells were defined as iDCs [29, 30]. The analysis was performed on BD Accuri™ C6 (BD Biosciences, USA), and the results were analyzed by BD Accuri™ C6 Plus System (BD Biosciences, USA). The proportions of mDCs and iDCs in peritoneal fluid samples were compared between endometriosis group, control group, as well as LPS group.

### Statistical analysis

GraphPad Prism Version 5.01 (GraphPad Software, San Diego, California, USA) was used for statistical analysis. Data were shown as mean ± standard error of the mean (SEM). Student’s *t*-test and oneway analysis of variance (ANOVA) were conducted respectively, to analyze the difference between groups and among groups. Data were shown as mean ± SEM. *P* value < 0.05 was considered statistically significant (^*^ *P* < 0.05, ^**^ *P* < 0.005 and ^***^ *P* < 0.001).

## Results

### Features of peritoneal DCs in endometriosis patients

**Fig. 1:**
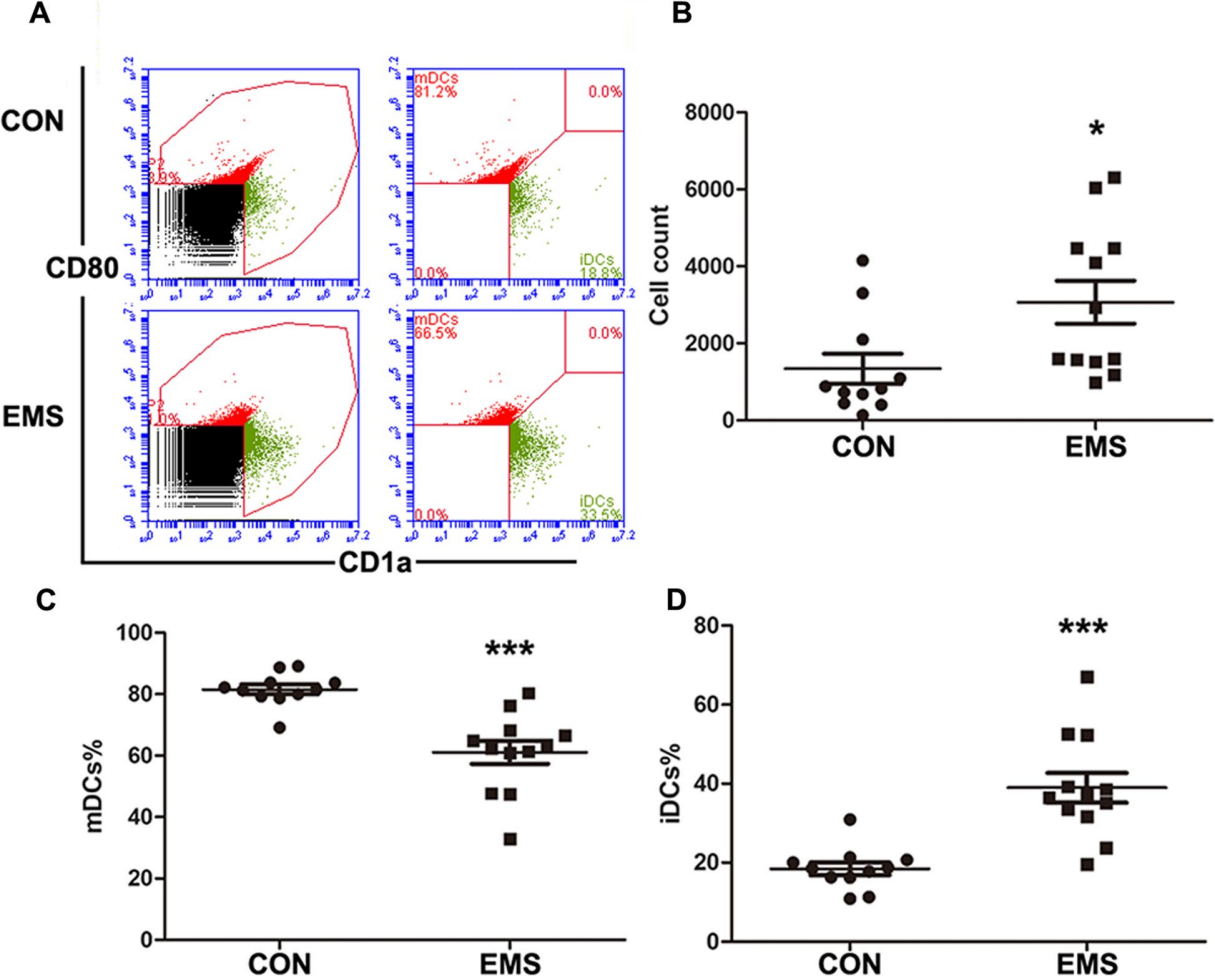
Features of peritoneal DCs in endometriosis patients. **A** Representative FACS analysis of peritoneal DCs in endometriosis group (EMS) and control group (CON). **B** Quantitative analysis of the number of peritoneal DCs in EMS and CON. **C** Quantitative analysis of the proportion of mDCs in EMS and CON. **D** Quantitative analysis of the proportion of iDCs in EMS and CON. (*P < 0.05 and ***P < 0.001)

The peritoneal DCs counts and proportion were identified by FACS analysis. As shown in Fig. 1, increased cell density of peritoneal DCs were observed in endometriosis patients. Moreover, FACS analysis demonstrated that the proportion of mDCs (CD80^high^CD1a^low^ cells) in the peritoneal DCs was lower in endometriosis patients. In contrast, the proportion of iDCs (CD80^low^CD1a^high^ cells) in the peritoneal DCs was increased in endometriosis patients.

### Dynamic changes of peritoneal DCs in the development of endometriosis

**Fig. 2:**
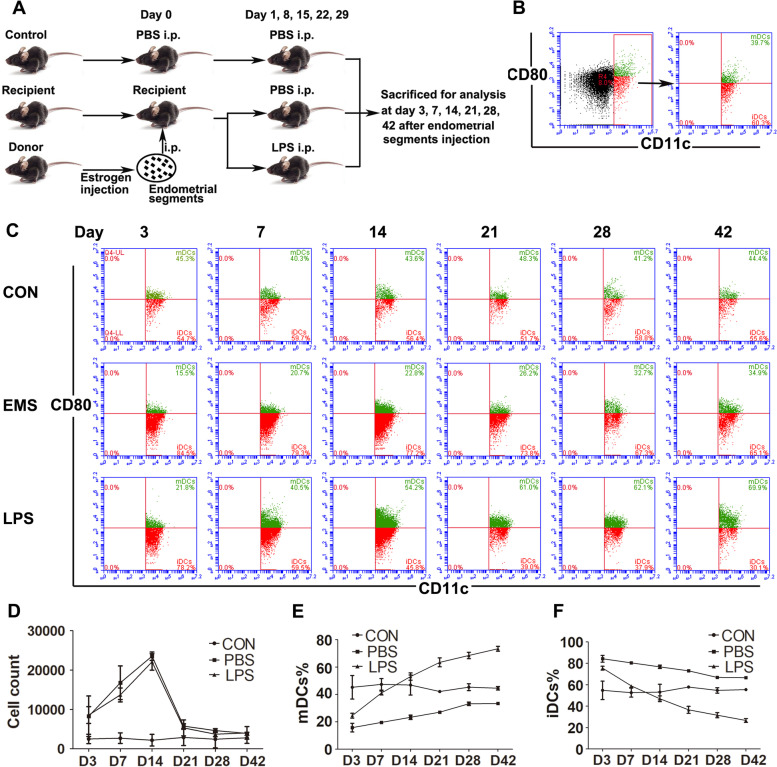
Features of peritoneal DCs in murine endometriosis models. **A** The flow diagram of the murine endometriosis models experimental design. **B** FACS analysis of the mDCs and iDCs in murine models. **C** Representative FACS analysis of dynamic changes of mDCs and iDCs in the development of endometriosis. **D** Quantitative analysis of the number of peritoneal DCs in the development of endometriosis. **E** Quantitative analysis of the proportion of mDCs in the development of endometriosis. **F** Quantitative analysis of the proportion of iDCs in the development of endometriosis

As shown in Fig. 2A, endometriosis models were induced by intraperitoneal injection of endometrial tissues to mimic endometriosis formation in humans. After intraperitoneal injection, the mice were sacrificed to evaluate the model formation at six time points (3, 7, 14, 21, 28 and 42 days). CD11c^high^ cells were marked as DCs. Among the DCs, mDCs express high level of CD80, while the iDCs express low level of CD80 (Fig. 2B). FACS analysis demonstrated that the cell density of peritoneal DCs increased immediately after the injection of endometrial tissues and reached the highest level at 14 days (Fig. 2C and D). In addition, the proportion of mDCs (CD11c^high^CD80^high^ cells) in the peritoneal DCs decreased immediately after the injection of endometrial tissues and then increased with the time until 42 days, but still lower than the control group (Fig. 2C and E). In contrast, the proportion of iDCs (CD11c^high^CD80^low^ cells) in the peritoneal DCs showed the opposite dynamic changes (Fig. 2C and F).

To assess the role of peritoneal DCs maturation in the progress of endometriosis, LPS was injected into the peritoneal cavity. The dynamic changes of peritoneal DCs cell density in the LPS group was consistent with the endometriosis group (Fig. 2C and D). Different from the endometriosis group, although the proportion of mDCs (CD11c^high^CD80^high^ cells) in the LPS group decreased immediately after the injection of endometrial tissues and increased with the time, the mDCs (CD11c^high^CD80^high^ cells) proportion was higher than the control group from 14 days after the injection (Fig. 2C and E). In consistent, the iDCs (CD11c^high^CD80^low^ cells) proportion in the LPS group showed the opposite dynamic changes (Fig. 2C and F).

### Relationship of peritoneal DCs and endometriosis

**Fig. 3:**
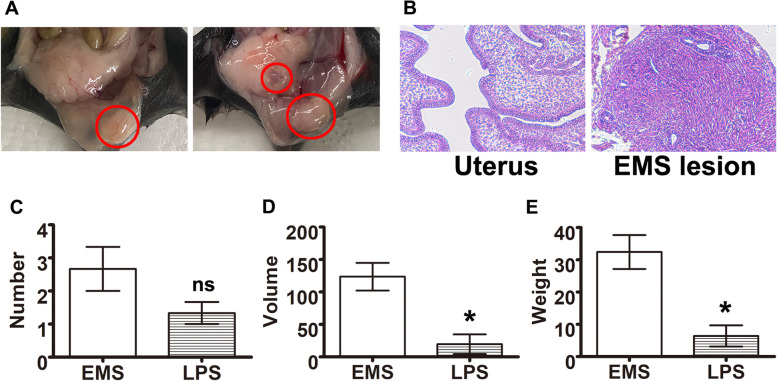
Ectopic lesions in murine endometriosis models. **A** Typical ectopic endometrial lesions in peritoneal cavity of endometriosis models. **B** HE staining of the eutopic and ectopic endometrial tissues. **C** Total number of lesions collected on Day 42 after modeling. **D** Total volume of lesions collected on Day 42 after modeling. **E** Total weight of lesions collected on Day 42 after modeling (*P < 0.05)

To verify whether peritoneal DCs maturation facilitate the progress of endometriosis, LPS was injected into peritoneal cavity to promote the maturation of iDCs. No significant change of body weight was observed after the peritoneal injection. The typical endometriotic lesions were observed 21 days in the peritoneal cavity after the injection in the endometriosis group and LPS group (Fig. 3A and B). The endometriosis lesions were collected at 42 days after the model induction. However, no significant difference was observed in the total number of lesions between the endometriosis group and the LPS group (Fig. 3C, *P* = 0.148). On the contrary, the total volume of the lesions in endometriosis group was significantly higher than that in the LPS group (Fig. 3D, *P* = 0.017). Similarly, the total lesion weight was also significantly increased in the endometriosis group (Fig. 3E, *P* = 0.014). The results revealed that after treated with LPS, the mDCs (CD11c^high^CD80^high^ cells) proportion was significantly increased, leading to lower volume and weight of the endometriosis lesions (Fig. 3D and E).

## Discussion

Our present findings provide new insights for understanding the role of peritoneal DCs in endometriosis. The research demonstrated an increased cell density of peritoneal DCs in endometriosis patients and murine models. However, the proportion of mDCs in the peritoneal DCs was lower in endometriosis patients. In addition, the proportion of mDCs in mouse models of endometriosis decreased immediately after the injection of endometrial tissues and then increased with the time until 42 days, but still lower than the control group. However, after treated with LPS, the mDCs proportion was significantly increased, leading to lower volume and weight of the endometriosis lesions.

Normally, retrograde endometrium should be cleared by activating immune cells and initiating an immune response in their ectopic environment. Therefore, menstrual reflux is only one of the causes of endometriosis, and abnormal immune function plays a key role in the survival and further development of endometrial lesions [31]. Immune tolerance is a necessary condition for the development of endometriosis. DCs are specialized in the initiation and modulation of the adaptive immune response. As effective stimulators of B and T lymphocytes, DCs can capture and process antigens, express lymphocyte co-stimulatory molecules, migrate to lymphoid organs and secrete cytokines to initiate immune response [32]. In addition, DCs can promote immune tolerance by negative selection of autoreactive T cells and generation of Tregs during the acquisition of central tolerance [33].

Depending on types of antigens, DCs could turn the immune response toward immunity or immune tolerance [34]. Suen et al. showed that the plasmacytoid DCs promoted endometriosis development through pathological angiogenesis during the early disease stage by secreting IL-10 [35]. After the DCs depletion, the size of endometriosis lesions was significantly reduced or increased in murine endometriosis models compared to control groups in which DCs were not ablated [20–22]. As for the peritoneal DCs, Guo et al. observed significant increase in the proportion of DC-like phenotype in peritoneal fluid [26]. Additionally, the proportion of myeloid DCs expressing mannose receptor was significantly higher in endometriosis tissues compared to the control group, promoting phagocytosis of dead endometrial cells and thereby contributing to the etiology of endometriosis [36]. In the present study, we observed an increased cell density of peritoneal DCs in endometriosis patients and murine models. The cell density of peritoneal DCs increased immediately after the injection of endometrial tissues and reached the highest level at 14 days. Thus, we assumed that the increased level of peritoneal DCs facilitated the pathogenesis of endometriosis lesions, especially in the early stage of the disease.

DCs maturation is the critical link between innate and adaptive T cell-dependent immunity [17]. As professional antigen-presenting cells, iDCs are characterized by strong migration ability, but lacking the ability to initiate T cell immune response, and may even induce immune tolerance. On the contrary, mDCs can effectively activate naive T cells, initiate T cell response, and play a central role in initiating, regulating and maintaining immune response [37]. Immature, migratory DCs loaded with tissue antigens are more effective at promoting peripheral tolerance in the steady state [38]. When it comes to endometriosis, the density of iDCs was significantly higher within the ectopic lesions in comparison to eutopic endometrium during the proliferative and secretory menstrual phases, while the mDCs were present in extremely low densities in ectopic lesions [25]. Tariverdian et al. evaluated leukocytes in the peritoneal fluid of women with endometriosis and revealed a moderate decrease of iDCs in patients with endometriosis, which became more profound with progressing disease, but did not reach levels of statistical significance [27]. Guo et al. evaluated the immune cells in peritoneal fluid using mass cytometry analysis and observed significant increase of iDCs in endometriosis patients compared to the control group [26]. Similarly, this research observed the decreased proportion of mDCs in the peritoneal DCs in endometriosis patients. In addition, endogenous DCs facilitated the growth and vascularization of endometriosis lesions, which enhanced endothelial cell migration by secreting proangiogenic factors [30]. As reported, iDCs shifted its immune role from presenting antigen to supporting angiogenesis and endometriosis progression, while DCs maturation suppressed this reaction [30]. In the present study, the proportion of mDCs in murine endometriosis models decreased immediately after the injection of endometrial tissues and then increased with the time until 42 days, but still lower than the control group. However, after treated with LPS, the mDCs proportion was significantly increased, leading to lower volume and weight of the endometriosis lesions. The results of this study indicated that DCs maturation played an important role in the development of endometriosis. The increased proportion of iDCs facilitated the development of endometriosis.

Taken together, peritoneal DCs maturation may be a potential new therapeutic target for endometriosis in the future. Although hormone therapy is effective, it requires long-term medication and has many side effects [39, 40]. The present research provided a new insight into the medical treatment for endometriosis. However, there are also some limitations. Firstly, the current study is limited by the small sample size. In addition, the mechanism of peritoneal DCs maturation in the development of endometriosis remains unclear. Studies with a larger sample size are necessary to confirm the results. Additionally, To identify the potential functions of peritoneal DCs maturation in pathogenesis of endometriosis, further investigations are required to explore the exact molecular mechanism, being our research strategies in further experiments.

## Conclusions

In conclusion, our study verified that increased level of peritoneal DCs facilitated the pathogenesis of endometriosis lesions, especially in the early stage of the disease. Furthermore, peritoneal DCs maturation played an important role in the development of endometriosis. This study is beneficial to further clarify the cause of endometriosis and provide a new therapeutic target for the treatment of endometriosis.

## Data Availability

The datasets used and analyzed during the current study are available from the corresponding author on reasonable request.

## Abbreviations

DCs: Dendritic cells
APCs: Antigen-presenting cells
iDCs: Immature DCs
mDCs: Mature DCs
peritoneal DCs: DCs in peritoneal fluid
FACS: Fluorescence associated cell sorting
PBS: Phosphatebuffered saline
LPS: Lipopolysaccharide
SEM: Standard error of the mean
ANOVA: Oneway analysis of variance
